# Validation of microsatellite multiplexes for parentage analysis and species discrimination in two hybridizing species of coral reef fish (*Plectropomus spp*., Serranidae)

**DOI:** 10.1002/ece3.1002

**Published:** 2014-04-24

**Authors:** Hugo B Harrison, Kevin A Feldheim, Geoffrey P Jones, Kayan Ma, Hicham Mansour, Sadhasivam Perumal, David H Williamson, Michael L Berumen

**Affiliations:** 1 Centre of Excellence for Coral Reef Studies, James Cook UniversityTownsville, Queensland, 4811, Australia; 2 Pritzker Laboratory for Molecular Systematics and Evolution, The Field Museum1400 S. Lake Shore Drive, Chicago, 60605, Illinois; 3 School of Marine and Tropical Biology, James Cook UniversityTownsville, Queensland, 4811, Australia; 4 Biosciences Core Laboratory, King Abdullah University of Science and Technology23955-6900, Thuwal, Saudi Arabia; 5 Red Sea Research Center, King Abdullah University of Science and Technology23955-6900, Thuwal, Saudi Arabia

**Keywords:** coral trout, hybridization, microsatellite multiplex, parentage analysis, *Plectropomus* spp

## Abstract

Microsatellites are often considered ideal markers to investigate ecological processes in animal populations. They are regularly used as genetic barcodes to identify species, individuals, and infer familial relationships. However, such applications are highly sensitive the number and diversity of microsatellite markers, which are also prone to error. Here, we propose a novel framework to assess the suitability of microsatellite datasets for parentage analysis and species discrimination in two closely related species of coral reef fish, *Plectropomus leopardus* and *P. maculatus* (Serranidae). Coral trout are important fisheries species throughout the Indo-Pacific region and have been shown to hybridize in parts of the Great Barrier Reef, Australia. We first describe the development of 25 microsatellite loci and their integration to three multiplex PCRs that co-amplify in both species. Using simulations, we demonstrate that the complete suite of markers provides appropriate power to discriminate between species, detect hybrid individuals, and resolve parent–offspring relationships in natural populations, with over 99.6% accuracy in parent–offspring assignments. The markers were also tested on seven additional species within the *Plectropomus* genus with polymorphism in 28–96% of loci. The multiplex PCRs developed here provide a reliable and cost-effective strategy to investigate evolutionary and ecological dynamics and will be broadly applicable in studies of wild populations and aquaculture brood stocks for these closely related fish species.

## Introduction

Microsatellite loci are commonly used in ecology to measure genetic variability within and among populations (Hartl and Clark 1989; Slatkin 1995). Their high allelic diversity and relative ease of development also make them ideal for individual genotyping to assist in species identification (Guichoux et al. 2011a), to uniquely identify individuals (Lukacs and Burnham 2005), and to infer phylogenetic or genealogical relationships (Blouin 2003; Jones et al. 2010). In the marine environment, these genetic tools may be the only means to measure important ecological processes such as larval dispersal (Planes et al. 2009; Saenz-Agudelo et al. 2011; Berumen et al. 2012), adult migrations (Hansen et al. 2001), and reproductive success (Araki et al. 2007; Beldade et al. 2012). However, applying these methods accurately can require numerous, highly polymorphic markers (Harrison et al. 2013a,b2013b), and optimized PCR multiplexes can maximize the cost-effectiveness of using microsatellites.

Coral trout (*Plectropomus,* Serranidae) are large predatory coral reef fishes that are widely distributed throughout the Indo-Pacific. They are among the highest market priced reef fish and often heavily exploited by both artisanal and commercial fisheries. The emergence of an international trade in live reef fish has significantly increased the demand for coral trout (Sadovy et al. 2003) with two of eight species in the genus now listed as “Vulnerable” in the IUCN species assessment (Sadovy de Mitcheson et al. 2013). On the Great Barrier Reef (GBR), *Plectropomus leopardus* (Lacepède, 1802) and *P. maculatus* (Bloch, 1790) are the most common coral trout species and frequently occur on the same reefs; however, their relative densities vary according to the cross-shelf position of individual reefs (Mapstone et al. 1998). Densities of *P. maculatus* are generally highest on inner-shelf reefs, while *P. leopardus* are at higher densities on mid- and outer-shelf reefs (Heemstra and Randall 1993; Mapstone et al. 1998; Russ et al. 2008). Adult *P. leopardus* and *P. maculatus* are easily identified in field observations by their characteristic spot patterns; however, the two species are not readily distinguishable as juveniles under approximately 60 mm in length. This poses a significant challenge in assessing the recruitment levels of *P. leopardus* and *P. maculatus* in areas where the two species co-occur. Furthermore, tank experiments have also shown that the two species can produce viable hybrid offspring (Frisch and van Herwerden 2006), and hybridization has led to genetic introgression in wild populations (van Herwerden et al. 2006).

In this study, we describe the development and validation of three multiplex PCR kits for individual barcoding and species discrimination of *P. leopardus* and *P. maculatus* DNA tissue samples, and for determining parent–offspring relationships in natural populations. Using an enriched cloning library developed for *P. maculatus* and 454 pyrosequencing libraries for both species, we identified and characterized 25 polymorphic microsatellite loci that amplified in multiplex PCR for both species. Using simulated datasets, we demonstrate the capacity of the marker set to discriminate between the two species, identify putative hybrid individuals, and resolve parent-offspring relationships in natural populations. Finally, we assess the transferability of each locus in seven species and subspecies in the genus *Plectropomus*.

## Material and Methods

### Sampling and DNA isolation

Tissue samples for 285 adult *Plectropomus leopardus* were collected from the Capricorn Bunker group (23°30′S, 152°50′E), a mid-shelf reef system of the southern Great Barrier Reef where *P. leopardus* and *P. maculatus* occur in sympatry. A further, 285 adult *P. maculatus* were collected from the Keppel Island group (23°10′S, 150°57′E), an inshore island group dominated by *P. maculatus*. All samples were collected between November 2007 and September 2012 under Marine Parks permit No. G06/17981.1 and G11/33554.1, and Queensland General Fisheries permit No. 87381 and 148534. Fish were captured using either baited, barbless hooks or on SCUBA with the use of biopsy probes attached to spears following Evans (2008). All individuals were identified to species level by trained observers and released alive at the capture site. Either fin or muscle tissue were removed from each fish and preserved in 95–100% ethanol. DNA extractions were performed from fish fin and muscle tissue using a Nucleospin-96 Tissue kit (Macherey-Nagel) with a double elution for final eluates of 200 *μ*L. Average DNA concentrations were 59.7 ng *μ*L^−1^ ± 8.9 SE for *P. leopardus* and 59.4 ng *μ*L^−1^ ± 5.5 SE for *P. maculatus*. In addition, samples of congeneric species were collected in order to examine cross-species amplification of microsatellite loci. These included 23 *P. areolatus* (Saudi Arabia), 24 *P. pessuliferus marisburi* (subspecies, Saudi Arabia), 29 *P. pessuliferus pessuliferus* (subspecies, Thailand and Maldives), 8 *P. oligacanthus* (Philippines), and 8 *P. laevis* (Maldives). Fin clips were removed from each fish and preserved in 95% ethanol. Total DNA was extracted using the QIAamp Tissue Kit (Qiagen, Germany), following the manufacturer's protocol.

### Microsatellite enrichment protocols

#### Cloning library

Microsatellite markers were developed for *P. maculatus* using an enrichment protocol developed by Glenn and Schable (2005). Approximately 4 mg of genomic DNA (gDNA) from one individual was digested with RsaI and XmnI, and SuperSNX24 linkers were ligated onto the ends of gDNA fragments. Linkers act as priming sites for polymerase chain reactions (PCRs) in subsequent steps. Five biotinylated tetranucleotide probes were hybridized to gDNA: (AAAT)_8_, (AACT)_8_, (AAGT)_8_, (ACAT)_8_, and (AGAT)_8_. The biotinylated probe-gDNA complex was added to magnetic beads coated with streptavidin (Dynabeads® M-280 Invitrogen, Carlsbad, USA). This mixture was washed twice with 2× SSC, 0.1% SDS and four times with 1×SSC, 0.1% SDS at 52°C. For the final two washes, the mixture was incubated for 1 min in a 52°C water bath. Between washes, a magnetic particle-collecting unit was used to capture the magnetic beads, which are bound to the biotin-gDNA complex. Enriched fragments were removed from the biotinylated probe by denaturing at 95°C and precipitated with 95% ethanol and 3 M sodium acetate.

To increase the amount of enriched fragments, a “recovery” PCR was performed in 25 *μ*L reactions containing 1× PCR buffer (10 mM Tris-HCl, 50 mM KCl, pH 8.3), 1.5 mM MgCl_2_, 0.16 mM of each dNTP, 10× BSA, 0.52 *μ*M of the SuperSNX24 forward primer, 1U *Taq* DNA polymerase, and approximately 25 ng enriched gDNA fragments. Thermal cycling was performed in an MJ Research DYAD as follows: 95°C for 2 min followed by 25 cycles of 95°C for 20 s, 60°C for 20 s, and 72°C for 90 s, and a final elongation step of 72°C for 30 min.

Subsequent PCR fragments were cloned using the TOPO-TA Cloning® kit (Invitrogen) following the manufacturer's protocol. Bacterial colonies containing a vector with gDNA (i.e., white colonies) were used as a template for subsequent PCR in a 25 *μ*L reaction containing 1× PCR buffer (10 mM Tris-HCl, 50 mM KCl, pH 8.3), 1.5 mM MgCl2, 0.12 mM of each dNTP, 10× BSA, 0.25 *μ*M of the M13 primers, and 1U *Taq* DNA polymerase. Thermal cycling was as follows: an initial denaturing step of 95°C for 7 min was followed by 35 cycles of 95°C for 20 s, 50°C for 20 s, and 72°C for 90 s, and PCR products were desalted using MultiScreen-PCR Filter Plates (Millipore, Billerica, USA) following the manufacturer's protocol. DNA sequencing was performed using the BigDye® Terminator v3.1 Cycle Sequencing Kit (Applied Biosystems, Foster City, USA). Sequencing reactions were precipitated with ethanol and 125 mM EDTA and run on an ABI 3730 DNA Analyzer.

## 454 pyrosequencing

454 pyrosequencing was performed on genomic DNA extracted from *Plectropomus leopardus* and *P. maculatus,* using the Genome Sequencer FLX, following the manufacturer's instructions (Roche 454 Life Sciences, Basel, Switzerland). Briefly, 500 ng of gDNA from each species was randomly sheared via nebulization, and double-stranded DNA adaptors were blunt-ligated to fragment ends using the GS FLX Titanium Rapid Library MID Adaptors Kit. The final single-stranded DNA library was isolated via magnetic streptavidin-coated beads binding to biotinylated adaptors. The library was then quantified via fluoro-spectrometer nanodrop 3300 (ThermoScientific, Wilmington, USA) and the size of the insert checked by 2100 Bioanalyser (Agilent biotechnologies, Inc, Santa Clara, USA) prior to emulsion PCR. Genomic shotgun library molecules were clonally amplified via emulsion PCR employing a GS FLX Titanium LV emPCR Kit. Following amplification, emPCRs were collected, and emulsions were broken. Beads containing sufficient copies of clonally amplified library fragments were selected via the specified enrichment procedure and counted with a Multisizer Coulter Counter (Beckman Coulter, Fullerton, USA) prior to sequencing. Following emulsion PCR enrichment, beads were deposited into the wells of a PicoTiterPlate device, and sequencing was performed. Image analysis, signal processing and base calling were performed using the Genome Sequencer FLX System Software supplied by 454 Life Sciences.

### Multiplex PCR development

#### Primer design

Microsatellite markers were isolated from two genomic libraries designed for *Plectropomus leopardus* and *P. maculatus*, and an enriched cloning library for *P. maculatus* of which, five loci were previously published in Harrison et al. (2012). Each library was screened for microsatellite loci containing uninterrupted tri- and tetra-nucleotide repeats with sufficiently large flanking regions for the design of oligonucleotide primers. Primers were designed in msatcommander (Rozen and Skaletsky 2000; Faircloth 2008) targeting 26 base pair oligonucleotides, melting temperatures of 60°C and 3′ G/C clamps for higher specificity. Tertiary structure formations were minimized between forward and reverse primers at each locus and between loci to reduce primer heteroduplexing in multiplex PCRs. In total, 46 markers were selected, 7 *P. leopardus* microsatellites, 14 and 25 *P. maculatus* microsatellites from the cloning library and genomic library, respectively. For each marker, the reverse primer was labeled with one of the fluorescent dies 6-FAM, HEX, Atto-550 or Atto-565.

All novel loci were amplified in simplex PCRs on three individuals from each species using the Invitrogen Platinum PCR kit with the following protocol. Each 20 *μ*L PCR contained, 2 *μ*L 10× buffer, 0.4 *μ*L dNTP (10 mM), 0.8 *μ*L MgCl (50 mM), 0.08 *μ*L HotStart Taq (5 U *μ*L^−1^), 2 *μ*L forward and reverse primers (2 *μ*M), 1 *μ*L genomic DNA and 11.72 *μ*L distilled water. PCRs were performed on Veriti thermal cyclers (Applied Biosystems) with the following sequence: 5 min initial denaturation at 95°C, 5 cycles of 30 s at 95°C, 30 s at 62°C, and 30 s at 72°C, then 5 cycles of 30 s at 95°C, 30 s at 60°C, and 30 s at 72°C, then 20 cycles of 30 s at 95°C, 30 s at 58°C, and 30 s at 72°C, followed by 10 min at 72°C. Touchdown PCRs greatly increase the binding specificity of primers and thus reduce noise and artifacts; these are commonly used for multiplex PCRs where optimal annealing temperatures of each locus differ. PCR products were screened on an ABI 3370xl DNA Analyzer (Applied Biosystems) following a 1:10 dilution. Markers that did not amplify in both species, with low-quality profiles (poor amplification or stutter), or with overlapping ranges were excluded from further experiments.

#### Multiplex PCR optimization

Microsatellites were selected for multiplex PCRs based on their likely size range, taking into account primer heteroduplexing. Selected primer pairs were combined in a primer premix for in-reaction concentrations ranging from 10 to 50 pM. Multiplex PCR amplification was performed on eight individuals of each species using the Qiagen Microsatellite Type-it kit (Qiagen, Germany). All three multiplex reactions were performed in a total volume of 10 *μ*L containing 5 *μ*L of Qiagen Multiplex Master Mix (2×), 3 *μ*L of distilled water, 1 *μ*L of primer premix, and 1 *μ*L template DNA. Multiplex PCRs were performed on Veriti thermal cyclers with the following sequence: 15 min initial denaturation at 95°C, 5 cycles of 30 s at 95°C, 90 s at 62°C, and 60 s at 72°C, then 5 cycles of 30 s at 95°C, 90 s at 60°C, and 60 s at 72°C, then 20 cycles of 30 s at 95°C, 90 s at 58°C, and 60 s at 72°C, followed by 30 min at 60°C. PCR products were screened on an ABI 3370xl DNA Analyzer (Applied Biosystems) with the GeneScan 500 or 600 LIZ (Applied Biosystems) internal size standard following a 1:15 dilution. The concentrations of primers were adjusted with each run for even amplification of all microsatellites. Individual genotypes were scored in genemapper v4.0, and unique alleles were distinguished using marker-specific binsets msatallele (Alberto 2009).

### Diversity and power analyses

For both *P. leopardus* and *P. maculatus*, observed genotypes were tested for departures from Hardy–Weinberg equilibrium due to heterozygote deficiency at each locus using the exact test (Guo and Thompson 1992) based on 1 000 000 Markov chain iterations as implemented in arlequin v3.5 (Excoffier and Lischer 2010). Significance of multiple *P*-values (*α* = 0.05) was assessed with strict Bonferroni correction applied for multiple comparisons (*P* < 0.001; Rice 1989). The number of alleles (*Na*), observed heterozygosity (*Ho*), expected heterozygosity (*He*), and the fixation index (*Fis*) were measured at each locus using genalex v6.5 (Peakall and Smouse 2012). The exclusion probability (*PE*) and cumulative exclusion probability were calculated according to Jamieson and Taylor (1997) in genalex v6.5. In order to estimate locus-specific genotypic error rates, 96 PCRs were repeated for each multiplex kit, which included 48 *P. leopardus* and 48 *P. maculatus* individuals.

### Species discrimination analysis

Using species-specific allelic frequency estimates, we simulated 5000 individuals for each species and 5000 F1 hybrid individuals in hybridlab v1.0 (Nielsen et al. 2006). We applied a model-based Bayesian clustering method implemented in structure 2.3.3 (Pritchard et al. 2000; Falush et al. 2007), using a Markov Chain Monte Carlo (MCMC) resampling procedure, to estimate the distribution of posterior probabilities of all 15 000 simulated individuals. With the number of groups (*K*) set to 2 corresponding to the two species and assuming population admixture, we performed a single run using 50 000 MCMC iterations with a burn-in period of 50 000 steps, first with each individual multiplex kit, and three combinations of multiplex kits combined. Following Vähä and Primmer (2006), we examined the distribution of posterior probabilities and identified species-specific thresholds of assignment to correctly allocate individuals to either species or hybrid individuals. The accuracy of assignments to purebred of hybrid groups was measured as the proportion of individuals in a group that were correctly identified.

### Accuracy of parentage analyses

Species-specific allelic frequencies were again used to determine the accuracy of parentage analyses in simulated populations of *P. leopardus* and *P. maculatus*. For each species, we generated 500 adult genotypes in the software program mykiss (Kalinowski 2009) with a sex ratio of two females per male, which approximates the observed sex ratio of *Plectropomus spp*. on inshore reefs of the Great Barrier Reef (Adams et al. 2000). We then generated 1000 offspring genotypes of known and unknown decent, simulating incomplete sampling of the adult populations. The proportion of known parents in the sample was fixed at 20%, and genotyping error was introduced at a rate of 1% for each locus.

Simulated datasets were analyzed using the pairwise likelihood score method implemented in famoz (Gerber et al. 2003). As the sex of individuals in wild-caught samples is generally unknown, our analyses combined simulated female and male genotypes. This approach accounts for genotyping error by introducing a calculation error rate when estimating the likelihood of each putative dyad. Here, a calculation error rate of 0.01% was used in all analyses (Gerber et al. 2000; Morrissey and Wilson 2005), with LOD score thresholds of 5 and 10 for single parents and parent pair assignments, respectively.

For each simulated offspring, the assigned parent or parent pair was compared with the known true parents. When an offspring was assigned to a parent that was not its true parent or not assigned (excluded), we determined whether the true parent was in the sample and identified it as either false-positive (type I) or false-negative (type II) errors (Fig. S1). The overall accuracy was measured as the proportion of correct assignments to single parents or parent pairs and the number of correct exclusions over all possible assignments (Harrison et al. 2013a). Assignments to a single parent when both parents were present in the sample were also considered as incorrect assignments (Fig. S1). Processing of all software outputs was performed in r with scripts uploaded as online supporting information.

## Results

### Multiplex PCR optimization

**Figure 1:**
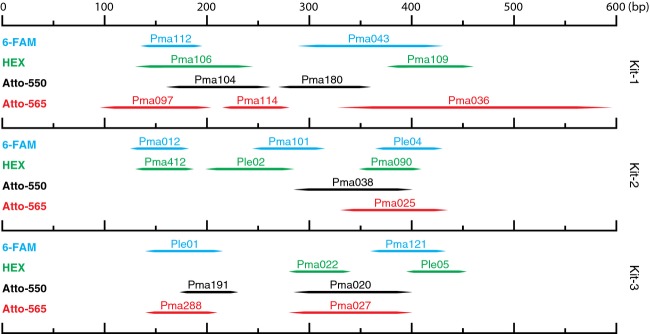
Allelic size ranges and fluorescent dyes used in each of three multiplex PCR kits designed for species discrimination and individual barcoding in natural populations of *P. leopardus* and *P. maculatus*. Horizontal axis shows the size ranges for each locus in base pairs (bp).

Among the 46 microsatellite loci isolated from either the enriched cloning library or the two genomic libraries for *P. leopardus* and *P. maculatus*, 21 were excluded due to incompatibility of product lengths (5 loci), low polymorphism (4 loci), poor amplification (6 loci), or because they did not amplify in one of the two species (6 loci). Of the 25 loci retained for multiplex PCRs, one contained tri-nucleotide (*Ple04*) and 24 contained tetra-nucleotide repeats (Table 1). All loci had good amplification qualities for both species, with sharp and even peaks standardized with variable primer concentrations. Loci covered an optimal range of product lengths allowing 8 or 9 loci per kit (Fig. 1) and additional loci may be included with further optimization.

**Table 1:** Description of 25 microsatellite loci isolated from a cloning and genomic libraries in *P. leopardus* and *P. maculatus*. The expected size range, fluorescent dye, and concentration of primers in PCRs and GenBank Accession Number is indicated for each locus

Locus	Forward primer (5′–3′)	Reverse primer (5′–3′)	GenBank accession no.	Library	Dye	Repeat motif	Reaction conc. (*μ*M)	Size range (bp)
***kit-1***								
Pma036^1^	GGGTCTGCAGGCAACACAAAGACAT	TGGAGAAAATTGTTGAGTGAAGAGTGG	JN222545	Cloning	Atto565	TAGA	0.040	328–596
Pma043	TGACTAACACTCAAATTGTCACCTTC	ATGCTGATAGGATGGTTTAATACAGC	KF992554	Cloning	6FAM	TATC	0.020	288–430
Pma097^1^	AGTGGGGCCATGTTTAACAACAGCA	ACGAGTTTTGTGAGATGGATGGGTGGA	JN222546	Cloning	Atto565	ATCT	0.010	96–206
Pma104^1^	CCATAACGGGGACTTTGGCCAATCA	CTGCACTTGTAGAACAGCCATGGGA	JN222547	Cloning	Atto550	TATC	0.010	160–266
Pma106^1^	CAGGAGCCATTGAGACAGGGAGAGG	AGTGTTGGTGGTTTCGCTGATGCTT	JN222548	Cloning	HEX	GATA	0.010	129–245
Pma109	TGCCCGACTCGATTTGTAACAGTGC	ACTCAGATATCTTGAGGTTAGAGGTC	KF992557	Cloning	HEX	ATCT	0.020	376–460
Pma112	CTGCACTTTAATACCCATGAAATAGC	TGGAAACCAGTTAAATAATCCCTGAC	KF992558	Cloning	6FAM	TATC	0.020	136–196
Pma114^1^	CTTGAACAGGCAGTGTAAAGGGGGC	ACCTGGAGCCAGTCATGTTCATGGT	JN222549	Cloning	Atto565	TATC	0.010	216–281
Pma180	AAATGGATATGACACAGAGATAGGAC	AATGAGAAGACATGTTGAAGCTGG	KF992560	Genomic	Atto550	AGAT	0.010	270–362
***kit-2***								
Ple002	TACTCGCAATTATAACACAGATCCAG	TTTGTCCAGCACTGTATTTATCTATC	KF992545	Genomic	HEX	AGAT	0.030	196–284
Ple004	ATTAGTATACAAGGAGCCACAGAATC	TCACTACGGCATTCCTAATAATTGTC	KF992546	Genomic	6FAM	AAT	0.030	366–430
Pma012	ATATGGCCATTATTGTGAGTTAGGTG	AAATCTTTAAACCTACCACTGATCCC	KF992548	Genomic	6FAM	AATG	0.010	128–182
Pma025	AGTAGACTCCGATAACTCATTCTCAC	TGAGACAAGAAGCTTTACAAGTGAAG	KF992551	Genomic	Atto565	ACTC	0.010	326–438
Pma038	TATGGAGGGATGATGCTATCTAAGAG	ATGCTAAACTGGATGCACTACAATC	KF992553	Genomic	Atto550	AGAT	0.010	286–400
Pma090	GATGTCCAAATATCACCTCTAACCAG	AGAGGCTCAATATTATCATGTGAACG	KF992555	Cloning	HEX	TAGA	0.010	352–408
Pma101	TGTTCTGTCAGATATGTAATGTGCTG	GGGGATAGACAAGAGGAAAGAGAGGGGA	KF992556	Cloning	6FAM	TATC	0.030	246–316
Pma412	AAAGTTAGCCATTTAAACACAGAGCC	TAGGTAGAGGTCACTGTTGCATTATC	KF992563	Genomic	HEX	ACAT	0.010	132–188
***kit-3***								
Ple001	CATCACTGATCACACTGCCTCC	AACCTTCACTACAGTTAATACCACAC	KF992544	Genomic	6FAM	ACGC	0.020	142–215
Ple005	AACTACAATGAAACCTGCCTCTTATG	TTTGATTATGACTCAATGATCGCAAG	KF992547	Genomic	HEX	ACAG	0.050	396–452
Pma020	TATGGAGGGATGATGCTATCTAAGAG	ATGCTAAACTGGATGCACTACAATC	KF992549	Genomic	Atto550	AGAT	0.015	285–399
Pma022	AAGATGTGCACTGTCAATACACTATG	GATGTCAGATATCAGGCTCCTAAATG	KF992550	Genomic	HEX	AGAT	0.020	278–340
Pma027	TAGACTAGTTCAGGGTGTCAGTTG	AAGGGAATGGAAATAAACTGTCATAC	KF992552	Genomic	Atto565	AAAT	0.015	280–394
Pma121	CTATTAGTTTCACTGAGGAAGAGTCG	ATATGAAGTTCACACCTCAGTTGAAC	KF992559	Cloningv	6FAM	ATAG	0.050	361–433
Pma191	GCCTTCGGAAACAATCATTATTCATC	GGGAAATTAAGAAGTCTACATTGAAGC	KF992561	Genomic	Atto550	AAAT	0.010	174–232
Pma288	TTGTATGTAATTTCGCCATGTTTGAG	TGTTGTCCGGTCATATTAATTGAGAG	KF992562	Genomic	Atto565	AAAG	0.010	140–210

1 Previously published in Harrison et al. (2012).

## Locus characteristics

We found no discernible differences in the quality and polymorphism of microsatellites developed from the cloning or genomic libraries. Among 285 *P. leopardus* and 285 *P. maculatus* individuals genotyped with all 25 loci, we found similar levels of genetic diversity in *P. leopardus* than in *P. maculatus* samples (Table 2). For *P. leopardus*, the mean number of alleles per locus was 17.9 ± 1.6 SE with an average observed heterozygosity of 0.723 ± 0.038 SE across all samples. For *P. maculatus*, the mean number of alleles per locus was 18.2 ± 2.2 SE with an average observed heterozygosity of 0.796 ± 0.019 SE across all samples. Several loci showed significant departure from Hardy–Weinberg equilibrium, though only one (*Pma112*) showed significant departure from expectations in both species. One locus, *Pma191*, was almost monomorphic in *P. leopardus* with one allele observed at a frequency of 0.98, but polymorphic in *P. maculatus*. The high level of polymorphism provide an average power of exclusion per locus of 0.463 ± 0.042 SE for *P. leopardus* and 0.479 ± 0.034 SE for *P. maculatus,* and each kit provided a cumulative power of exclusion of ∼0.99 (Table 2). The overall combined power of exclusion with 25 loci was approximately 1 – 2 × 10^−8^. Scoring errors in repeated PCR samples were rare, and error rates were estimated from the combined *P. leopardus* and *P. maculatus* genotypes (Table 2). Only *Pma112* exhibited high levels of scoring error, which were due to the presence of null alleles.

**Table 2:** Characteristics of three multiplex kits based on 285 *P. leopardus* and 285 *P. maculatus* individuals

	*Plectropomus leopardus* (*N* = 285)							*Plectropomus maculatus* (*N* = 285)							
			
Locus	Na	Ho	He	Fis	HWE *P* value	PE	CumPE	Na	Ho	He	Fis	HWE *P* value	PE	CumPE	Scoring error, %
***kit-1***															
Pma036	43	0.800	0.934	0.144	**<0.001**	0.768	0.768	59	0.961	0.957	−0.005	0.742	0.842	0.842	1
Pma043	20	0.800	0.787	−0.017	0.953	0.419	0.865	20	0.807	0.839	0.038	0.092	0.536	0.927	0
Pma097	17	0.853	0.857	0.005	**<0.001**	0.553	0.940	25	0.888	0.887	−0.001	0.651	0.638	0.973	0
Pma104	20	0.772	0.782	0.013	0.993	0.439	0.966	40	0.912	0.924	0.013	0.812	0.736	0.993	0
Pma106	25	0.902	0.928	0.029	0.893	0.747	0.991	17	0.839	0.871	0.038	0.927	0.595	0.997	0
Pma109	31	0.614	0.950	0.354	**<0.001**	0.817	0.998	17	0.811	0.807	−0.004	0.452	0.480	0.999	1
Pma112	15	0.607	0.854	0.289	**<0.001**	0.555	0.999	14	0.646	0.785	0.178	**<0.001**	0.422	0.999	8
Pma114	7	0.551	0.532	−0.036	0.198	0.146	0.999	16	0.828	0.815	−0.016	0.012	0.489	1.000	0
Pma180	19	0.758	0.777	0.024	0.497	0.439	1.000	18	0.751	0.759	0.010	0.368	0.415	1.000	0
***kit-2***															
Ple002	21	0.877	0.868	−0.010	0.278	0.587	0.587	14	0.860	0.854	−0.007	0.965	0.548	0.548	0
Ple004	16	0.747	0.759	0.015	0.754	0.402	0.753	11	0.744	0.740	−0.005	0.172	0.350	0.706	0
Pma012	14	0.867	0.854	−0.015	0.256	0.541	0.887	10	0.681	0.716	0.049	0.361	0.318	0.800	0
Pma025	19	0.765	0.774	0.012	0.991	0.426	0.935	22	0.902	0.908	0.007	0.651	0.686	0.937	0
Pma038	23	0.891	0.882	−0.010	0.409	0.619	0.975	20	0.916	0.891	−0.027	0.743	0.649	0.978	0
Pma090	10	0.460	0.472	0.025	0.391	0.113	0.978	10	0.758	0.740	−0.025	0.429	0.358	0.986	0
Pma101	10	0.691	0.728	0.051	0.375	0.329	0.985	15	0.709	0.683	−0.038	0.460	0.305	0.990	0
Pma412	9	0.632	0.657	0.039	0.147	0.270	0.989	9	0.656	0.640	−0.025	0.546	0.246	0.993	0
***kit-3***															
Ple001	21	0.853	0.903	0.055	0.411	0.673	0.673	20	0.793	0.765	−0.036	0.736	0.416	0.416	1
Ple005	14	0.782	0.808	0.032	0.077	0.467	0.826	8	0.698	0.662	−0.055	0.820	0.240	0.556	0
Pma020	23	0.895	0.883	−0.014	0.510	0.620	0.934	20	0.916	0.892	−0.027	0.758	0.649	0.844	0
Pma022	14	0.600	0.616	0.027	0.934	0.237	0.949	9	0.740	0.741	0.001	0.903	0.358	0.900	0
Pma027	23	0.895	0.865	−0.034	0.730	0.586	0.979	25	0.839	0.858	0.023	0.970	0.569	0.957	0
Pma121	15	0.825	0.875	0.057	0.256	0.594	0.992	15	0.796	0.760	−0.048	0.060	0.384	0.973	0
Pma191	10	0.042	0.042	−0.012	1.000	0.001	0.992	12	0.856	0.859	0.003	0.313	0.555	0.988	0
Pma288	9	0.604	0.630	0.042	0.020	0.224	0.993	10	0.604	0.567	−0.065	0.586	0.180	0.990	0

*N*, number of individuals genotyped; Na, total number of alleles; Ho, observed heterozygosity; He, expected heterozygosity; Fis, fixation index; HWE, exact test for Hardy–Weinberg Equilibrium; PE, Probability of Exclusion; CumPE, Cumulative Probability of Exclusion for each multiplex PCR.

Significant departure from HWE are indicated in bold.

### Species discrimination

**Figure 2:**
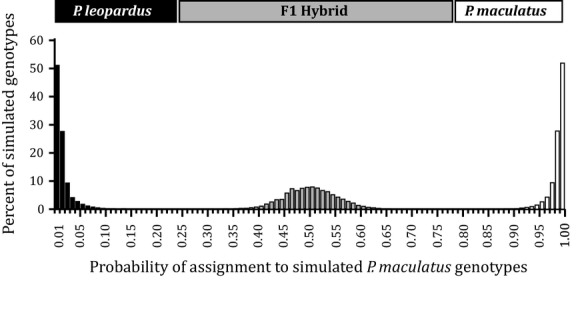
Distribution of the posterior probability of assignment for 5000 simulated *P. leopardus* genotypes, 5000 *P. maculatus* genotypes, and 5000 interspecific F1 hybrids as determined in structure with 25 microsatellite loci.

Assignment tests on 15 000 simulated genotypes successfully distinguished between *P. leopardus*, *P. maculatus,* and interspecific hybrids with a high degree of confidence. Combining all 3 multiplex kits, each group was clearly delimitated resulting in 100% confidence in assignments (Fig. 2). Independently, multiplex kits still resolved each group with ∼99% confidence (Table 3). Assignment thresholds were based on the posterior probability of assignment to the *P. leopardus* group and set *ad lib* to minimize the overall number of errors. Individuals with a posterior probability of assignment ≤0.240 were identified as *P. maculatus*, >0.780 were *P. leopardus* and between 0.240 and 0.780 were identified as interspecific hybrids. Assignment thresholds can be modified to meet study-specific objectives, for example maximizing the accuracy of species identification only. Furthermore, if the aim is to discriminate *P. leopardus* from *P. maculatus*, a single multiplex kit can provide sufficient power. Multiplex kit-3 provided to most discriminatory power of any single kit due to the presence of *Pma191*, which is almost monomorphic in the sampled population of *P. leopardus*.

**Table 3:** Proportion of incorrect assignments of simulated genotypes with optimum intervals of 0–0.24 (*P. leopardus*), 0.25–0.78 (F1 hybrids), and 0.79–1 (*P. maculatus*) with single and combined multiplex kits

Kit	No. loci	*P*. *leopardus*	F1 hybrids	*P*. *maculatus*
*kit-1*	9	0.22	1.15	0.16
*kit-2*	8	0.12	0.86	0.16
*kit-3*	8	0.14	1.33	0.08
*kits 1 & 2*	17	0	0	0
*kits 2 & 3*	16	0	0	0
*kits 1, 2 & 3*	25	0	0	0

### Accuracy of parentage

By analyzing simulated datasets with known parent–offspring relationships, we were able to measure the accuracy of parentage analyses and identify the frequency of type I (false positive) and type II (false negative) assignment errors. Combining all three multiplex kits with an introduced error rate of 1% per locus, we were able to correctly assign or correctly exclude 99.8 ± 0.1% SE of *P. leopardus* and 99.6 ± 0.1% of *P. maculatus* offspring. All assignment errors were due to genotypic mismatches in the simulated datasets. Two types of errors were identified at low frequency (Table 4): in order of abundance, these included i) wrongly assigning an offspring to a single parent when neither of the true parents were in the sample; and ii) assigning an offspring to a parent pair, where one parent was correctly assigned and the other falsely assigned when the true parent was present in the sample. Increasing LOD thresholds may not reduce the rate of Type I error because all errors were caused by simulated genotyping error.

**Table 4:** Accuracy of parent–offspring assignment in simulated populations of *P. leopardus* and *P. maculatus* based on species-specific allelic frequencies

	*P. leopardus*				*P. maculatus*			
		
	*sim-1*	*sim-2*	*sim-3*	Average	*sim-1*	*sim-2*	*sim-3*	Average
Accuracy of								
Single parent assignments	0.997	0.997	0.997	0.997	1	1	1	1.000
Assignments to parent pairs	1	1	1	1.000	1	1	1	1.000
Exclusions	1	0.999	0.997	0.998	0.992	0.992	0.997	0.994
False-positive rate (Type I)	0.001	0.002	0.003	0.002	0.005	0.005	0.002	0.004
False-negative rate (Type II)	0	0	0	0.000	0	0	0	0.000
Overall accuracy	0.999	0.998	0.997	0.998	0.995	0.995	0.998	0.996

### Marker transferability

All but one species (*P. punctatus*) within the genus *Plectropomus* (Serranidae) were tested for the transferability of loci developed here for *P. leopardus* and *P. maculatus*. The overall transferability rate of loci across congeneric species ranged from 28 to 96%, notwithstanding monomorphic loci (Table 5). Given the small sample sizes for some species, it is possible that not all true alleles were observed. Of the 25 markers developed, 25 successfully amplified in samples of *P*. *pessuliferus pessuliferus*, 19 in *P. areolatus*, 17 in *P. pessuliferus marisburi*, 16 in *P. laevis*, and 14 in *P. oligocanthus*.

**Table 5:** Transferability of microsatellite multiplex PCRs within the genus *Plectropomus*

Species Sampling location	*P. areolatus* Thuwal, Saudi Arabia			*P. pessuliferus marisburi* Thuwal, Saudi Arabia			*P. pessuliferus pessuliferus* Maldives (*N* = 9) & Thailand (*N* = 20)			*P. oligacanthus* Bohol, Philippines			*P. laevis* Maldives		
					
Locus	*N*	Na	Range (bp)	*N*	Na	Range (bp)	*N*	Na	Range (bp)	*N*	Na	Range (bp)	*N*	Na	Range (bp)
*kit-1*															
Pma036	21	13	520–616	22	15	458–550	29	16	456–536	7	7	522–594	6	8	495–551
Pma043							26	15	356–424						
Pma097	23	5	94–118	22	7	102–125	29	4	86–104	8	2	94–102	8	1	86
Pma104							29	8	177–209						
Pma106	22	8	158–198	19	11	174–215	28	12	157–199	8	4	145–161	8	4	200–212
Pma109	17	9	394–446	10	7	348–419	26	8	358–406						
Pma112	18	5	176–192				26	3	148–160						
Pma114	23	6	252–272	24	5	218–228	29	3	220–228	8	1	219	8	1	219
Pma180	22	12	277–337	21	7	277–334	29	10	293–329	8	5	333–358	8	5	292–308
*kit-2*															
Ple002	21	10	217–261	16	10	244–292	28	12	207–263						
Ple004							29	4	384–394						
Pma012	23	5	132–148	23	6	116–152	29	12	132–178	8	3	128–136	8	4	132–148
Pma025	23	1	236	24	3	234–238	29	8	336–368	8	1	236	8	1	236
Pma038	23	11	304–344	23	9	281–319	29	9	280–324	4	5	305–329	7	2	281–297
Pma090	23	9	385–417				29	2	365–369						
Pma101	23	2	251–255	24	3	248–256	29	7	252–298	6	2	250–274	7	1	252
Pma412	20	1	153	14	4	149–165	29	5	153–173	8	2	139–143	8	3	153–161
*kit-3*															
Ple001							29	5	150–178						
Ple005							27	6	406–430						
Pma020	22	11	304–344	19	7	281–317	28	8	296–324				5	1	282
Pma022	22	1	291	21	4	283–295	29	4	283–303	5	2	283–291	5	1	291
Pma027	19	11	294–342	16	2	304–308	28	10	308–348	8	6	328–344	8	4	308–332
Pma121	18	10	383–463	14	14	388–472	27	9	379–419				4	5	379–415
Pma191	20	2	179–183	12	1	183	27	1	183	8	1	183	8	1	183
Pma288							27	4	148–164	8	7	162–198	8	1	133

*N*, number of individulals genotyped; *Na*, total number of alleles.

## Discussion

The three multiplex PCR kits developed herein allow fast, accurate, and cost-effective genotyping of individuals of two closely related species of coral trout: *Plectropomus leopardus* and *P. maculatus*. Each kit is composed of a selection of 8 or 9 highly polymorphic microsatellite loci that, independently, provide confident identification of each species and interspecific hybrid individuals. When combined, the three kits accurately also identified parent–offspring relationships with over 99.6% accuracy, providing unprecedented resolution of individual barcoding for these highly valued species. Given the high transferability of the markers among species, the PCR kits will be useful for investigating a range of population and evolutionary processes in this important genus.

Investigating life-history processes in coral reef fishes can be challenging and technical advances in both the isolation of molecular markers and high throughput screening of multilocus genotypes have introduced new tools to ecologists tackling questions that were once intractable (Gardner et al. 2011). *P. leopardus* and *P. maculatus,* two important fishery species, have a complex and intertwined evolutionary history (van Herwerden et al. 2006). This novel set of microsatellite loci provides a strong basis to investigate whether contemporary hybridization is occurring in mixed populations of *P. leopardus* and *P. maculatus* throughout the Great Barrier Reef. As juveniles are morphologically indistinguishable, it also provides a simple assay to examine early life-history processes that are important in determining the distribution, abundance, and fishery stocks of these species. However, assignment thresholds for each class of individuals are likely to depend on the degree of introgression of sampled populations. Where the repeated backcrossing of interspecific hybrids with either parent species has resulted in multiple hybrid categories, other approaches that directly estimate the probability of individuals belonging to each category (Anderson and Thompson 2002) may be more appropriate. As such, simulations should always be performed with study-specific allele frequencies for each species.

Recent work has already demonstrated the potential of parentage analysis in coral reef fish to provide invaluable insight into the reproductive success and juvenile dispersal of *Plectropomus spp*. (Harrison et al. 2012; Almany et al. 2013). When combined, the multiplex kits developed here provide unprecedented accuracy in the resolution of parent–offspring relationships in natural populations, minimizing both false-negative and false-positive assignments (Harrison et al. 2013a,b2013b). These kits may also be used to infer other genealogical relationships, determine pedigrees in aquaculture brood stocks, and provide insight into the heritability of desirable traits for aquaculture.

Many experimental methods are effective for screening microsatellite (Gardner et al. 2011; Guichoux et al. 2011b). Overall, we found no discernible differences in the quality of markers identified from either cloning libraries or 454 pyrosequencing. However, the high throughput of next generation sequencing technologies greatly facilitated the optimization of multiplex PCRs by avoiding primer incompatibilities. Aside from one locus showing high levels of genotyping error (*Pma112*) in both species, the overall marker set of 25 loci demonstrated remarkably high specificity in not only *P. leopardus* and *P. maculatus*, but also other species in the genus.

In conclusion, the genetic tools developed here will provide the means to answer a broad range of ecological and evolutionary questions for this important genus of groupers. Globally, serranids are one of the most important fishery species on coral reefs, and they represent important apex predators that are increasingly threatened by overexploitation (Sadovy de Mitcheson et al. 2013). The ability to discriminate species, identify individuals, and determine parent–offspring relationships will greatly aid in the development of both sustainable harvesting and conservation measures.

## Data Accessibility

Novel DNA sequences: GenBank Accessions KF992544 – KF992563. R scripts: uploaded as online supporting information with example data.

## Supporting Information

**Figure S1.** Decision tree that lead to correct and incorrect assignments in parentage analysis.

**Data S1.** R scripts and example data to measure the accuracy of parentage analysis for simulated parent and offspring genotypes.

**Data S2.** Example offspring file of all possible outcomes of a parentage test.

**Data S3.** Example result file of a parentage analysis with all possible decision outcomes.
