# Supplementary material for: Validation of microsatellite multiplexes for parentage analysis and species discrimination in two hybridizing species of coral reef fish (Plectropomus spp., Serranidae)
Source: Ecol Evol. 2014 Apr 24;4(11):2046–57. doi: 10.1002/ece3.1002 (PMC4201420; doi:10.1002/ece3.1002)
Supplement: Supplementary file 1 — Figure S1. Decision tree that lead to correct and incorrect assignments in parentage analysis. [file ece30004-2046-sd1.docx]

**Supporting information**

**Validation of microsatellite multiplexes for parentage analysis and species discrimination in two hybridising species of coral reef fish (*Plectropomus spp.*, Serranidae)**

Hugo B Harrison^1,*^, Kevin A Feldheim^2^, Geoffrey P Jones^1,3^, Kayan Ma^3^, Hicham Mansour^4^, Sadhasivam Perumal^4^, David H Williamson^1^, Michael L Berumen^5^

**Figure S1** Decision tree that lead to correct and incorrect assignments in parentage analysis. There are only three correct decisions in parentage analyses, assigning the true parent or true parents when one or both parents are in the sample (correct assignment) and assigning no parent when thee true parent is not in the sample (true exclusion). Assignment errors are either false positive (Type I) or false negative (Type II) depending on whether the true parent or parents are present in the sample. Simulations can identify the susceptibility of a given marker set to different types of error. The overall accuracy is the sum of all errors over the total number of possible assignments.
